# Evidence of a strong coupling between root exudation, C and N availability, and stimulated SOM decomposition caused by rhizosphere priming effects

**DOI:** 10.1002/ece3.311

**Published:** 2012-07-05

**Authors:** Per Bengtson, Jason Barker, Sue J Grayston

**Affiliations:** 1 Department of Biology – Microbial Ecology, Lund UniversitySölvegatan 37, 223 62, Lund, Sweden; 2 Department of Forest Sciences, University of British ColumbiaVancouver, British Columbia, V6T 1Z4, Canada

**Keywords:** Carbon sequestration, coupled biogeochemical cycles, elevated CO _2_, global warming, microbial C assimilation, nitrogen mineralization, plant–microbial feedbacks, soil respiration

## Abstract

Increased temperatures and concomitant changes in vegetation patterns are expected to dramatically alter the functioning of northern ecosystems over the next few decades. Predicting the ecosystem response to such a shift in climate and vegetation is complicated by the lack of knowledge about the links between aboveground biota and belowground process rates. Current models suggest that increasing temperatures and rising concentrations of atmospheric CO_2_ will be partly mitigated by elevated C sequestration in plant biomass and soil. However, empirical evidence does not always support this assumption, as elevated temperature and CO_2_ concentrations also accelerate the belowground C flux, in many cases extending to increased decomposition of soil organic matter (SOM) and ultimately resulting in decreased soil C stocks. The mechanism behind the increase has remained largely unknown, but it has been suggested that priming might be the causative agent. Here, we provide quantitative evidence of a strong coupling between root exudation, SOM decomposition, and release of plant available N caused by rhizosphere priming effects. As plants tend to increase belowground C allocation with increased temperatures and CO_2_ concentrations, priming effects need to be considered in our long-term analysis of soil C budgets in a changing environment. The extent of priming seems to be intimately linked to resource availability, as shifts in the stoichiometric nutrient demands of plants and microorganisms will lead to either cooperation (resulting in priming) or competition (no priming will occur). The findings lead us on the way to resolve the varying response of primary production, SOM decomposition, and release of plant available N to elevated temperatures, CO_2_ concentrations, and N availability.

## Introduction

Increased temperatures and concomitant changes in vegetation patterns are expected to dramatically alter the functioning of northern ecosystems over the next few decades (Joos et al. 2001; Euskirchen et al. 2009). Predicting the ecosystem response to such a shift in climate and vegetation is complicated by the lack of knowledge about belowground processes. Current models suggest that increasing temperatures and rising concentrations of atmospheric CO_2_ will be partly mitigated by elevated C sequestration in plant biomass and soil (Joos et al. 2001; Gerber et al. 2004). However, empirical evidence does not always support this assumption. Elevated CO_2_ and warming experiments show that the effect vary widely between either prolonged, temporary, or no stimulation of plant growth (Reich et al. 2006), and also that enhanced decomposition of soil organic matter (SOM) might result in decreased soil C stocks (Kirschbaum 1995; Macdonald et al. 2011). Possible reasons for the discrepancy between theory and empirical data are the paucity of real data, such that current theories and models do not accurately address three major factors of importance for C cycling and sequestration: (1) links between aboveground biota and belowground process rates; (2) the stoichiometric coupling of C and N cycles; and (3) priming effects.

Organisms require C and N in strict proportions and couple the cycling of these elements by assimilating and synthesizing compounds with specific C/N ratios. Thus, it is not possible to fully understand the turnover of C and N without considering the ways in which they both constrain each other's behavior (Reich et al. 2006; Högberg et al. 2010). For example, C sequestration is regulated by the biogeochemical cycling of N, such that N limits primary production in most ecosystems (Vitousek and Howarth 1991). At the same time, plant roots provide exuded C that stimulate the activity of microorganisms that decompose SOM and release plant available N (Fontaine et al. 2004, 2007; Fontaine and Barot 2005). However, the continuous supply of root exudates turns the rhizosphere into an environment with high numbers of microbes and high microbial activity (Norton et al. 1990; Norton and Firestone 1991). In such microenvironments, nutrients other than C, mostly N, may be limiting microbial growth (Cheng et al. 1996), resulting in competition for N between plants and soil microorganisms (Schimel et al. 1989; Zak et al. 1990; Månsson et al. 2009). On the other hand, the exuded C might cause “priming” of SOM, leading to increased decomposition of SOM and release of plant available N (Dijkstra et al. 2009). The factors determining the net outcome between these opposing processes are not well known, and their relative importance for determining plant N availability remains uncertain.

Priming is defined as an increase in decomposition of SOM in response to the input of easily available C or N sources (Blagodatskaya and Kuzyakov 2008). Real priming needs to be distinguished from apparent priming, as apparent priming results from an increased C and N mineralization in response to a higher turnover rate of the microbial biomass, without an accompanying increase in SOM decomposition. Failing to accurately differentiate between real and apparent priming has implications for our interpretation of changing below and aboveground C inputs in a changing climate. If recent belowground plant C inputs are quantitatively important in stimulating microbial growth and driving microbial immobilization of N (by presenting microbes with a more easily degradable source of C than SOM [apparent priming]), the potential for increased C fixation in plants as a consequence of elevated CO_2_ levels may not be realized in N-limited ecosystems. On the other hand, if the continuous input of root exudates results in increased SOM decomposition and release of plant available N, plant growth and C sequestration might increase. Therefore, assigning increased SOM turnover to real or apparent priming effects results in directly opposing interpretations and predictions regarding C sequestration and mineralization in a changing climate.

The mechanism causing priming remains elusive. Most proposed explanations suggest that the additional C is causing an increase in the metabolism and growth of microbial r-strategists followed by the emergence of a second population of K-strategists, ultimately leading to SOM decomposition (Fontaine et al. 2004; Blagodatskaya and Kuzyakov 2008). The proposed mechanism is likely relevant under pulsed inputs of high concentrations of C, such as cellulose in straw and litter. It is harder to see how the dynamics between r- and K-strategists would work in a system with semicontinuous input of low concentrations of easily available C. The rhizosphere represents such an environment (Hinsinger et al. 2009), and to our knowledge, there is no evidence to suggest that the microbial community in the rhizosphere is characterized by cyclic variations in the relative abundance of microbial r- and K-strategists.

The advent of stable isotope analysis makes it possible to study priming effects in environments receiving semicontinuous inputs of C, such as the rhizosphere, as well as to distinguishing between real and apparent priming. By pulse-labeling plants with ^13^CO_2_ and tracing the ^13^C into the soil, and combining this with the ^15^N pool-dilution method to simultaneously estimate gross N transformation rates, we can estimate belowground C allocation, quantify microbial C and N assimilation, and identify the C sources used for microbial growth.

The objective of this experiment was to identify links between plant C exudation and belowground C and N turnover, with the aim of increasing our understanding of the factors regulating SOM decomposition and release of plant available N. This was achieved by examining the effect of different tree species belowground C inputs (root exudates) on soil C and N turnover. The experiment was designed to test two alternative hypotheses, namely that exuded C result in (1) apparent priming or (2) real priming.

## Material and Methods

### Experimental design

**Figure 1:**
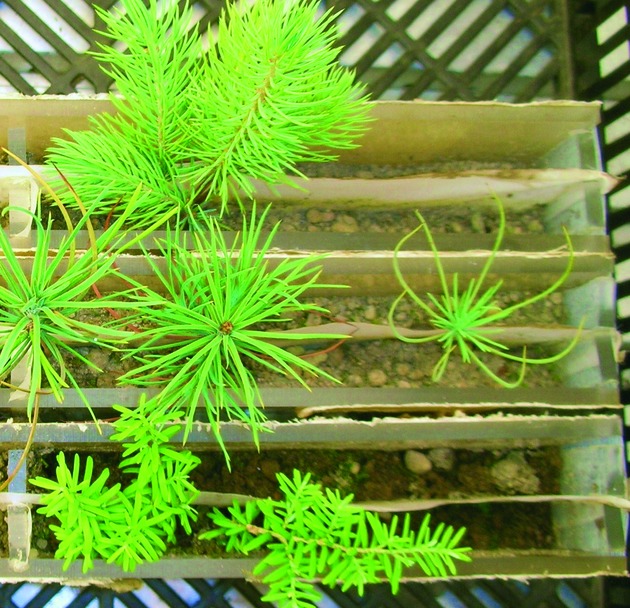
Experimental system: ponderosa pine (*Pinus ponderosa*), Sitka spruce (*Picea sitchensis*), and western hemlock (*Tsuga heterophylla*) seedlings were grown in separate Plexiglas boxes. The boxes were divided lengthwise into two compartments using a membrane (pore size 0.45 *μ*m) that allowed free movement of the soil solution containing root exudates, but excluded plant roots and mycorrhizal hyphae. Each compartment measured 147 × 12 × 150 mm (B × D × H) and contained approximately 210 g of soil.

The two alternative hypotheses were tested in an experiment where ponderosa pine (*Pinus ponderosa*), Sitka spruce (*Picea sitchensis*), and western hemlock (*Tsuga heterophylla*) seedlings were grown in separate boxes. Each box was divided lengthwise using a membrane into two compartments: one containing the plant and its roots and the second excluded roots but still allowed for free movement of the soil solution containing the root exudates (Fig. 1). By using three different tree species and dividing the boxes into one compartment with roots and one without roots, a range of root exudation rates was achieved. Root exudation rates were estimated in a ^13^CO_2_ pulse-chase experiment, and the gross N mineralization and assimilation rates were estimated using the ^15^N pool-dilution method. The quantification and separation of priming effects into real or apparent priming were done by relating the rate of SOM decomposition and microbial C assimilation to measured root exudation rates. Apparent priming would result in a ratio between microbial C assimilation to root exudation of one or less (depending on the microbial C-use efficiency). On the other hand, real priming would cause each exuded C molecule to result in a disproportionally high SOM decomposition and microbial C assimilation, inferring that low inputs of root exudates invoke a quantitatively larger release of C from SOM.

### Cultivation of seedlings

Soil was collected from the forest in Pacific Spirit Park, close to the University of British Columbia, Vancouver, British Columbia. The forest is located in the coastal western hemlock (CWH) biogeoclimatic zone (Pojar et al. 1986). The dominating trees (approximately 100 years old) were western red cedar (*Thuja plicata*), western hemlock (*T. heterophylla*), and Douglas-fir (*Pseudotsuga menziesii*). Forest floor material was removed and mineral soil collected with a shovel to a depth of 20–25 cm. Stones and coarse roots were removed and large soil aggregates (>1 cm) broken up. The soil was then stored at 4°C until the start of the experiment. The soil was a ferro-humic podzol, pH was around 5.5, and the organic matter content was 5% (measured by loss on ignition). Stratified seeds of ponderosa pine, Sitka spruce, and western hemlock were germinated and grown in separate Plexiglas boxes containing the Pacific Spirit Park soil. The boxes were divided lengthwise into two compartments using a membrane that allowed free movement of the soil solution containing root exudates, but excluded plant roots and mycorrhizal hyphae (pore size 0.45 *μ*m, Plastok Ltd, Merseyside, UK). Each compartment measured 147 × 12 × 150 mm (B × D × H) and contained approximately 210 g of soil kept at 20% moisture (by weight) content. The boxes were kept in growth chambers at a temperature of 22°C and a light–dark cycle of 12 h. There were six replicates of each box and two to three seedlings of the same tree species in one box.

### Determination of root exudation rates

The rate of root exudation was estimated in a ^13^C pulse-chase experiment performed sixth months after the seedlings had germinated. The boxes containing the seedlings were placed in Plexiglas containers (volume 7.5 L) with a removable tight-fitting lid. The containers were sealed with vacuum grease and Parafilm M, and 30 mL ^13^C-enriched CO_2_ (99 atom% ^13^C, Cambridge Isotope Laboratories, Andover, Massachusetts) injected through a butyl rubber septa. The seedlings were incubated in the ^13^CO_2_ enriched atmosphere for 3 h in the same growth chambers. Six days after the pulse labeling, the boxes were taken apart and all soil in the two compartments collected. After careful removal of plant roots, the soil was thoroughly mixed and then immediately freeze-dried and stored frozen until further analysis. The concentration of SOM and its *δ*^13^C_PDB_ (the residual ^13^C remaining in soil 6 days after labeling) was determined by grinding freeze-dried soil samples in a Retch MM200 ball mill (ball diameter: 5 mm; Retsch GmbH, Haan, Germany) at max speed for 2 min. A portion of the soil was then transferred to a 5 × 8 mm tin cup, and analyzed by PDZ Europa 20/20 continuous flow Isotope Ratio Mass Spectrometer (CF-IRMS), connected to a gas/solid/liquid preparation module (ANCA-GSL, PDZ Europa Scientific Instruments, Crewe, UK).

Trees exude recently fixed C within 1–4 days (Ekblad and Högberg 2001). Given the size of the seedlings used in this experiment, a 6-day chase period should be enough to ensure exudation of the ^13^C-labeled photosynthates. The ^13^C recovered in the soil thus represented the proportion of photosynthate that was produced and exuded during 3 h of photosynthesis, and the rate of root exudation was calculated from the ^13^C recovered in soil by equation 1:


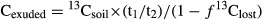
(1)

where C_exuded_ is the rate of the supply of root exudates to the different compartments in the microcosms (mg C kg^−1^ d^−1^), ^13^C_soil_ is the measured soil concentration of ^13^C (mg kg^−1^ above background), *f*^13^C_lost_ is the fraction of exuded C that is lost from the, for example, CO_2_ (see below for a description of this term), t_1_ is the length of the light period (hours), and t_2_ is the duration of the labeling period (hours).

### Estimation of gross N transformation rates

Microbial N assimilation, nitrification, and mineralization were determined by the ^15^N pool-dilution method in the same boxes that was used for the ^13^CO_2_ pulse labeling described above. Five days after the ^13^CO_2_ pulse labeling, 20 mL of a solution containing 2.4 *μ*g ^15^NH_4_^+^-N mL^−1^ (as ^15^NH_4_Cl, 98% ^15^N, Cambridge Isotopic Laboratories) were added by syringe to the soil in the boxes. The solution was injected at 8–10 locations within each compartment to ensure an even distribution of the label. Within 2 h after the addition of the ^15^NH_4_^+^-label, one set of boxes were destructively sampled and approximately 10 g of soil extracted with 1.0 mol/L KCl. The amount of ^14^NH_4_^+^-N, ^15^NH_4_^+^-N, ^14^NO_3_^−^-N, and ^15^NO_3_^−^-N in the extract was determined by IRMS after diffusion, according to standard procedures (IAEA 2001). Another set of boxes (the boxes that was sampled for ^13^C-analysis) were harvested 24 h after addition of the ^15^NH_4_^+^ solution (i.e., 6 days after the ^13^CO_2_ pulse labeling) and treated as above. Gross N mineralization, nitrification, and total and microbial assimilation of N were calculated from the differences in concentration and ^15^N content of NH_4_^+^, NO_3_^−^, and organic N between the samples taken 2 and 24 h after the addition of the label using FLUAZ (Mary et al. 1998). The calculations assume that the gross N transformation rates remained constant and that no ^15^N was recycled to the enriched pool during the measurement period. The short assay period endeavored to fulfill this assumption.

### Evaluation and quantification of priming effects

Decomposition of SOM in response to root exudates was calculated from equations 2–4. First, the microbial C assimilation was calculated. Microbial assimilation of native SOM is notoriously hard to estimate, and we therefore calculated the total microbial C assimilation from the microbial N assimilation as in Bengtson and Bengtsson (2007):


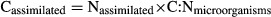
(2)

where C_assimilated_ is the microbial C assimilation rate, N_assimilated_ is the microbial N assimilation rate, and C:N_microorganisms_ is the average C:N ratio of the soil microorganisms.

The total microbial C demand (i.e., the sum of microbial assimilation and respiration, corresponding to the decomposition of SOM) needed to support the C assimilation was then calculated according to equation 3:


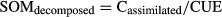
(3)

where SOM_decomposed_ is the total SOM decomposition (in C units) and CUE is the microbial C-use efficiency.

**Figure 2:**
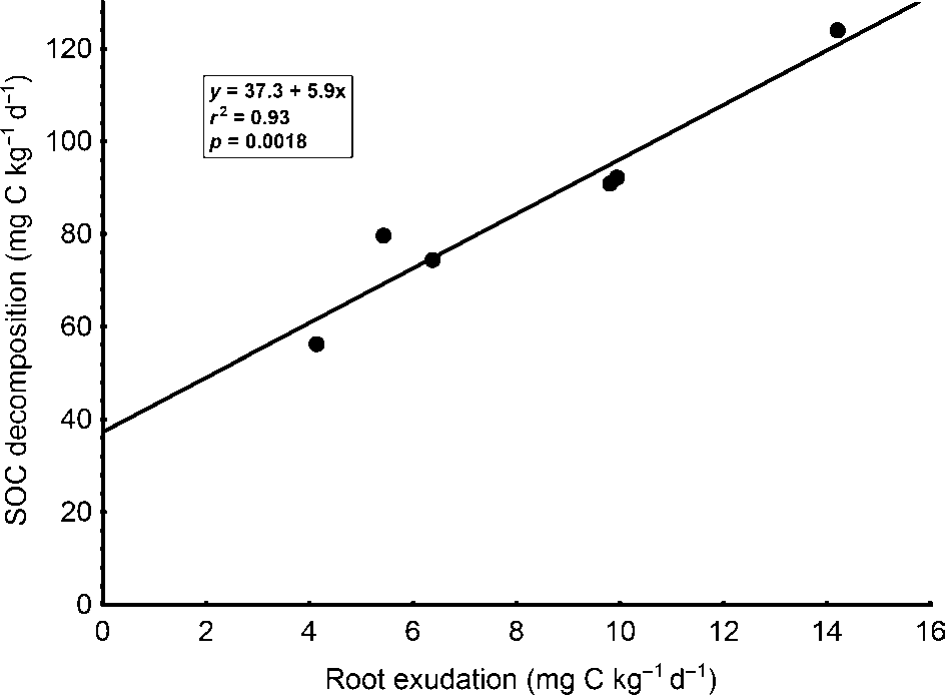
The relationship between root exudation and SOM decomposition.

Finally, the decomposition of SOM that could be assigned to priming (SOM_primed_) at different root exudation rates was calculated from equation 4:


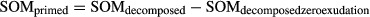
(4)

where SOM_decomposed_ is the observed SOM decomposition and SOM_decomposed zero exudation_ is the intercept of the correlation between root exudation and SOM_decomposed_ (Fig. 2), that is, the SOM decomposition in the absence of root exudation.

To test the robustness of the deterministic calculations of SOM decomposition and priming effects by equations 1–4, a probabilistic Monte Carlo analysis was performed in @RISK 5.7 (Palisade Corporation, New York). Mean and standard deviation of the variables in equations 1–4 were used as input, and the distributions truncated at the 99% upper and lower confidence limits. The Latin Hypercube technique was used to sample the probability distributions, and 100,000 simulations were preformed. An in-built multiple linear regression analysis was employed to help identify which of the input distributions contributed most to the variation in the output distribution.

Three of the factors in equation 1–4 were based on literature values rather than being measured. For all three of these factors, we used conservative estimates with broad probability distributions, in an attempt to ensure that we did not overestimate priming effects. (1) The microbial C:N ratio is remarkably stable among soils and ecosystems, with an average of 8.6 (Cleveland and Liptzin 2007). On the contrary, there is a huge degree of variation in reported conversion factors (ranging from 0.13 to 0.92) within and among soils when the chloroform-fumigation technique is used to estimate microbial C and N (Joergensen et al. 2011). Rather than introducing this uncertainty in our calculations, we allowed the C:N ratio of the microbial biomass to vary between 6.6 (representing a microbial community dominated by bacteria) and 10.6 (representing a microbial community almost completely dominated by fungi), with an average value of 8.6. (2) Our experimental setup did not allow us to measure heterotrophic soil respiration rates in each treatment separately. The loss of ^13^C from the respective treatment was therefore calculated from the published, observed recovery of ^14^C after spiking a wide range of soils with low concentrations of, for example, ^14^C-labeled organic acids, sugars, and other compounds representative of root exudates. The recovery of ^14^C in such studies usually varies between 65% and 85% of the added ^14^C (Boddy et al. 2008; Rousk et al. 2011), irrespective of type of compounds, soil type, and soil pH. Based on these findings, we allowed the recovery of ^13^C to vary between 0.5 and 0.9 in our calculations, with an average value of 0.7 (calculated from original data, Rousk et al. 2011, on the average recovery of 12 different ^14^C-labeled compounds 172 h after addition to soil). (3) Determining the C-use efficiency of soil microorganisms remains a challenge. Even if we have the means to determine variations in the relative growth rate of bacteria and fungi among soils and treatments with precision, there is a high degree of uncertainty when using conversion factors to calculate the absolute rate of production of fungal and bacterial biomass (Rousk and Bååth 2011). Bengtson and Bengtsson (2007) used a different approach based on the stoichiometric coupling of microbial C and N demands to demonstrate that the average C-use efficiency in the soil environment is approximately 0.4, which is well within the range normally found for fungi and bacteria (Holland and Coleman 1987). Based on these studies, we varied the C-use efficiency in our calculations between 0.3 and 0.5, with a mean of 0.4. It should be noted that several studies that have reported C-use efficiencies below 0.3. However, as an underestimation of the C-use efficiency would lead us to overestimate priming effects, a lower limit of 0.3 is suitable for the purpose of this study.

### Validation of the calculations and assumptions

We used three different criteria to validate our calculations and assumptions: (1) The calculated SOM decomposition should be correlated with actual measurements of gross N mineralization, a proxy for SOM decomposition (e.g., Bengtson et al. 2005 and references therein). (2) As there was no or very little N loss from the soil in our experimental system (apart from plant uptake), the C:N ratio of the biomass growing on the decomposed C should not be significantly different from the C:N ratio used in our calculations. (3) The estimated SOM decomposition and priming effects should be more dependent on the measured factors compared with the factors that were assumed (i.e., C-use efficiency, the microbial C:N ratio, and the recovery of ^13^C). To test this criteria, an in-built multiple linear regression analysis was employed in @RISK to help identify which of the input distributions contributed most to the variation in the output distributions.

## Results

**Figure 3:**
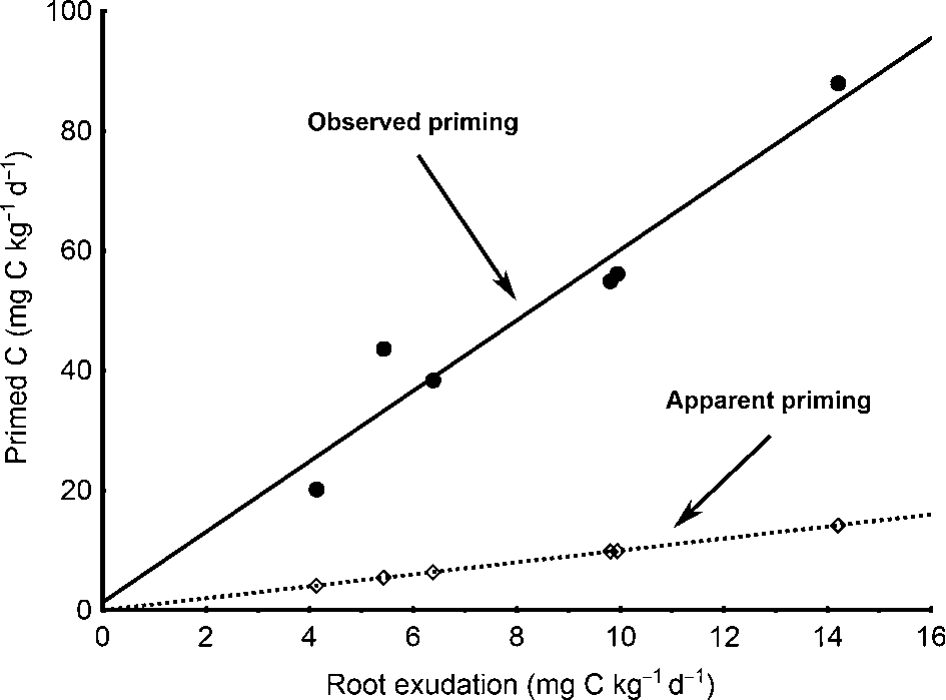
The relationship between root exudation and SOM decomposition caused by rhizosphere priming. The solid line represents the priming that was observed in our experiment, and the dotted line the maximum “apparent priming” that could result from direct stimulation of microbial growth by the exuded C.

The decomposition of SOM varied between 56 and 124 mg C kg^−1^ d^−1^ (Table 1), and there was a strong positive correlation between the rate of root exudation and the rate of SOM decomposition (Fig. 2). We can therefore conclude that priming occurred in our experimental system. The steep slope of the regression line suggests that the priming effect caused by the exuded C was much stronger than can be explained a direct stimulation of microbial growth (i.e., apparent priming) (Fig. 3). In fact, each milligram of exuded C resulted in decomposition and release of 6 mg bioavailable C from SOM (Fig. 2). Therefore, our first hypothesis that exuded C result in apparent priming was rejected, and the hypothesis that root exudation results in real priming (increased SOM decomposition) accepted.

**Table 1:** The rate of root exudation, microbial C and N assimilation, gross N mineralization, total SOM decomposition, and the SOM decomposition that can be assigned to priming in the different treatments

	Ponderosa pine		Sitka spruce		Western hemlock	
			
	No roots	Roots	No roots	Roots	No roots	Roots
C_exuded_	5.43 (0.84)	9.94 (1.69)	4.13 (0.65)	14.2 (3.1)	6.38 (0.88)	9.80 (1.51)
N_assimilated_	3.67 (0.33)	4.25 (0.39)	2.59 (0.91)	5.72 (0.54)	3.43 (0.32)	4.19 (0.38)
C_assimilated_	31.6 (4.1)	36.6 (4.8)	22.3 (8.1)	49.2 (6.5)	29.5 (3.9)	36.0 (4.7)
N_min_	4.21 (0.39)	4.58 (0.43)	2.73 (0.26)	6.20 (0.58)	3.64 (0.34)	4.52 (0.36)
SOM_decomposed_	79.6 (12.9)	92.2 (15.0)	56.2 (21.3)	124.0 (20.3)	74.4 (12.2)	90.9 (14.8)
SOM_primed_	43.6 (12.0)	56.2 (11.9)	20.2 (3.9)	88.0 (13.3)	38.4 (12.0)	54.9 (12.0)
Priming (%)	121	156	56	244	107	152
SOM_primed_: SOM_decomposed_ (%)	54.8	60.9	36.0	71.0	51.6	60.4

All rates are in mg kg^−1^ dw soil d^−1^ and abbreviations are the same as in Material and Methods section. Values between brackets represent the standard deviation of the mean (standard deviation of the mean for C_exuded_, C_assimilated_, SOM_decomposed_, and SOM_primed_ were derived from the Monte Carlo analysis).

**Figure 4:**
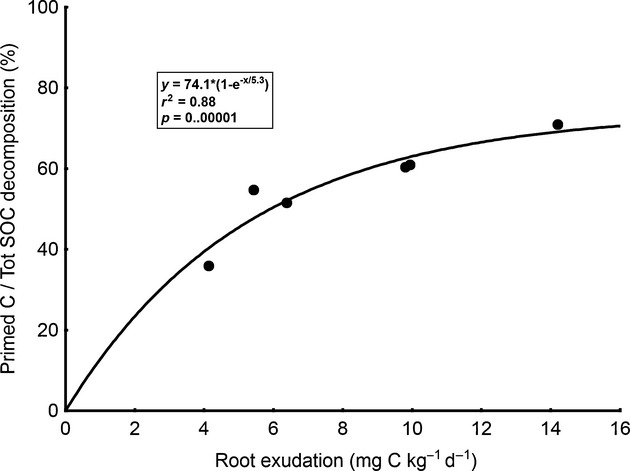
The fraction (%) of the total SOM decomposition that can be assigned to priming at different root exudation rates. There is no or little additional increase in the relative importance of priming for SOM decomposition at root exudation rates exceeding 16 mg C kg^−1^ soil d^−1^ (see text for details).

The priming effect resulted in an increase in SOM decomposition of between 56% and 244% (Table 1), while the fraction of the total SOM decomposition that could be assigned to priming varied between 36% at the lowest exudation and 70% at the highest exudation (Table 1). The relative importance of priming for total SOM decomposition seemed to reach an asymptote at high root exudation rates (Fig. 4). Based on this observation, we determined the maximum possible priming that could potentially occur in our system and at which root exudation rate this occurred by fitting an exponential function to our data according to Equation 5:


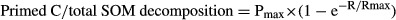
(5)

where P_max_ is the maximum fraction of SOM decomposition that can be caused by priming, R is the root exudation rate, and 3 × R_max_ is the root exudation rate at which the fraction of SOM decomposition that is caused by priming is less than 5% from of the asymptote (P_max_). The analysis suggests that there is no or little additional increase in the relative importance of priming for SOM decomposition at root exudation rates exceeding 16 mg C kg^−1^ soil d^−1^. It should be noted that none of the compartments in our microcosms received exuded C at this rate, and it cannot be excluded that the relative importance of priming for SOM decomposition decreases rather than reaching an asymptote at such high root exudation rates.

**Figure 5:**
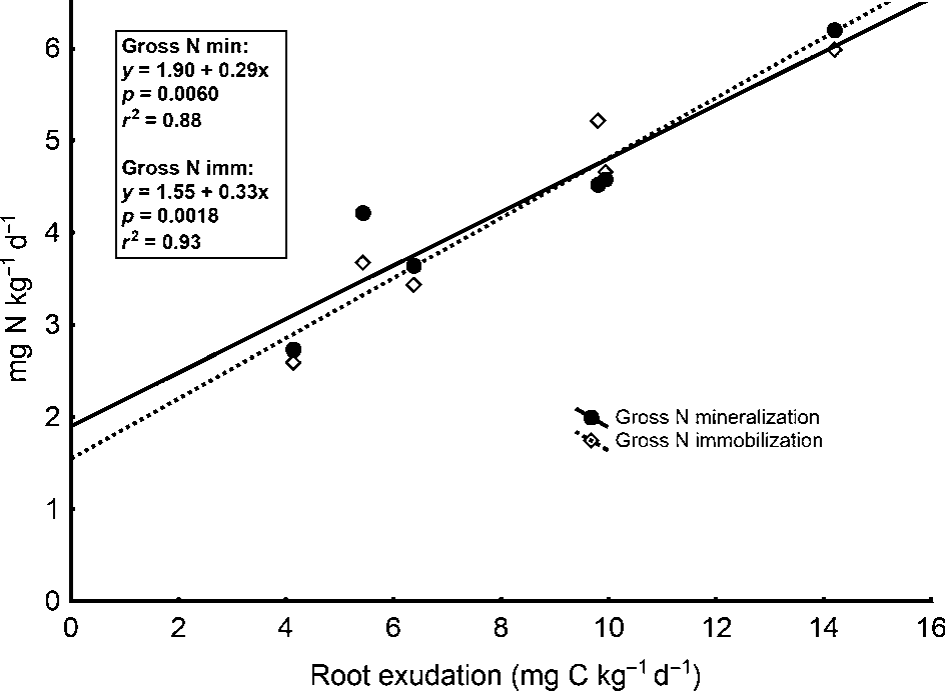
The relationship between root exudation and gross N mineralization and immobilization.

**Figure 6:**
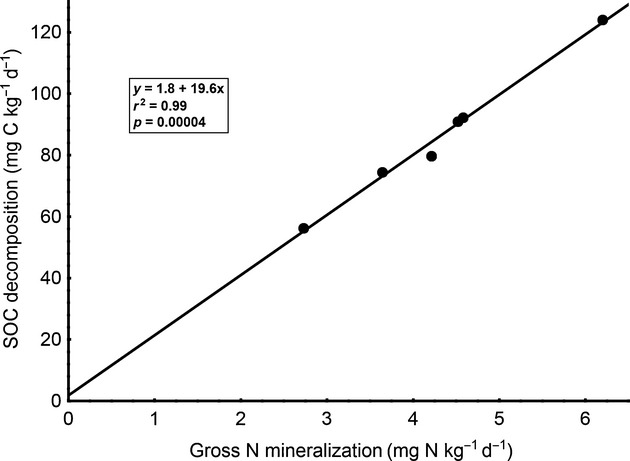
The relationship between gross N mineralization and SOM decomposition.

The response of gross N mineralization and immobilization to root exudates was similar to that of SOM decomposition (Fig. 5). At high supply rates of root exudates, the release of plant available N was strongly enhanced, while the effect was less apparent at low root exudation rates. On average, 0.3 mg N was mineralized for each milligram of exuded C (Fig. 5). There was also a strong correlation between gross N mineralization and the calculated SOM decomposition (Fig. 6). The first criterion for accepting our calculations that the calculated SOM decomposition should be correlated with actual measurements of gross N mineralization was, therefore, fulfilled. The slope of the regression line between gross N mineralization and SOM decomposition revealed that the decomposed SOM had a C/N ratio of 20 (Fig. 6). The newly formed biomass growing on the decomposed SOM had a C/N ratio of 7.8, when calculated using the same C-use efficiency as in Equation 3 (data not shown). This corresponds well with the estimated C/N ratio of the microorganisms used in Equation 2 (6.6–10.1). Thus, the C:N ratio of the biomass growing on the decomposed C was not significantly different from the C:N ratio used in our calculations of SOM decomposition and priming effects, and therefore, our second criterion for accepting our calculations was fulfilled.

Gross N immobilization was the factor measured that had the largest influence on the calculated SOM decomposition and priming effects, and the estimated SOM decomposition and priming effects were thus more dependent on the measured factors, rather than on the factors that were based on literature values. All three criteria set up to test the validity of our calculations were therefore fulfilled and the results robust regarding the assumptions.

## Discussion

The substantial stimulation of SOM decomposition in response to root exudates has implications for our long-term understanding of soil C stocks in a changing climate. Plants generally exude more C at high temperatures and CO_2_ levels (Macdonald et al. 2011; Phillips et al. 2011). If these exudates are quantitatively important in driving microbial growth and assimilation of N (Hart et al. 1994), the potential for increased C fixation in plants due to elevated temperatures and CO_2_ levels would not be realized in N-limited ecosystems. Our results suggest this is not the case. On the contrary, increased root exudation rates resulted in a significant increase in the decomposition of SOM, and equally important, a concurrent increase in the release of plant available N. Thus, even if priming occurs to the extent that was observed in this study, it cannot be assumed that the enhanced SOM decomposition will result in decreased soil C stocks. The combination of increased temperatures, elevated CO_2_ concentrations, and elevated release of plant available N might stimulate primary production and ecosystem C sequestration to the extent that it fully compensates for the increased SOM decomposition (Graaff et al. 2006; Dijkstra et al. 2008; Zak et al. 2011).

Accordingly, we found a strong connection between root exudation and gross N mineralization, suggesting that plants became progressively less N-limited at high root exudation rates. Furthermore, the relative importance of stimulated SOM decomposition by priming seemed to reach an asymptote at high root exudation rates, and a possible explanation to these findings is that as N availability increases, plants allocate more C to aboveground net primary production (Phillips et al. 2009, 2011; Högberg et al. 2010). On the contrary, N deficient plants increase C allocation belowground. Plants would thus be expected to exude C that stimulated SOM decomposition and N mineralization when they are strongly N-limited, and there are observations that the effect is more pronounced at elevated CO_2_ concentrations (Dijkstra et al. 2008; Phillips et al. 2009, 2011; Zak et al. 2011).

In terrestrial ecosystems, release of C by decomposition of SOM generally represents the rate limiting step of microbial growth (Bengtson and Bengtsson 2007; Demoling et al. 2007). The decomposition results in a concurrent release of plant available N and is governed by oxidative enzymes such as peroxidases (Osono 2007; Hofrichter et al. 2010; Drake et al. 2011). The activity of such enzymes is in turn dependent on the availability H_2_O_2_ produced by various oxidases that use simple C sources such as sugars, organic acids, and alcohols as substrate (Ander and Marzullo 1997; Halliwell and Gutteridge 1999). If plants provide these C sources through root exudation, the cost of using such enzyme systems to degrade SOM decreases, as implicitly suggested by our observation of a positive relationship between root exudation, SOM decomposition, and N mineralization. However, at high root exudation rates, soil microorganisms might directly assimilate the exuded C and decrease the production of exoenzymes used to decompose SOM. This would drive the microbial community toward N limitation, resulting in competition for N between plants and microorganisms and decreased priming effects, as observed in soils receiving a pulsed input of large quantities of C (Blagodatskaya and Kuzyakov 2008). Due to the limited range of root exudation rates in our experiment, we cannot exclude that even higher exudation rates would have resulted in similar results. In fact, at the highest root exudation rate in our experiment, the total N immobilization by plants and soil microorganisms exceeded the amount of N released from SOM (Fig. 5).

Taken together, N-limited plants and C-limited microorganisms appear to be a prerequisite for priming to occur. However, if the ecosystem is N-poor to the degree that the activity of decomposer microorganisms is N-limited as well, there seem to be a tipping point where no priming would occur. Accordingly, when temperate and boreal forests are fertilized with N or exposed to high N deposition rates, SOM decomposition and soil respiration generally decrease and soil C stocks increase (Janssens et al. 2010; Liu and Greaver 2010). Severely N-limited ecosystems such as the arctic tundra seem to respond in the opposite way. When these soils are fertilized with N soil, SOM decomposition increases, resulting in decreased soil C stocks (Mack et al. 2004). The combined response to warming, N fertilization, and elevated CO_2_ seems to be site specific (Macdonald et al. 2011), with results from CO_2_ enrichment experiments vary widely between no, temporary, and prolonged stimulation of gross primary production, and also between no, increased, and decreased SOM decomposition (Reich et al. 2006). Our observations provide a possible explanation to the contradictory results and suggest that extent of priming might vary among ecosystems due to variations in resource availability, as also proposed by Milcu et al. (2011).

Our findings that rhizosphere priming effects caused an increase in SOM decomposition of 56–244% are remarkable but still reflective of previous studies, which have found priming to cause an increase in SOM mineralization of up to 360% (Blagodatskaya and Kuzyakov 2008; Dijkstra and Cheng #b^501^; Kuzyakov #b^502^). In fact, it is possible that our choice of methodology might have slightly underestimated the priming effect. For example, it cannot be excluded that some of the ^13^C recovered in soil 6 days after the labeling of plants was contained inside mycorrhizal hyphae, leading to an overestimation of root exudation rates. Since the mycorrhizal hyphae were restricted to the compartment containing the plant and its roots, this would result in an even more pronounced dependency of SOM decomposition on root exudates. Furthermore, we used conservative estimates with broad probability distributions for the microbial C-use efficiency (compare, e.g., Holland and Coleman 1987) and recovery of ^13^C (compare, e.g., Boddy et al. 2008; Rousk et al. 2011) when estimating the root exudation rates, possibly leading to a slight overestimation of root exudation, and consequently, slightly underestimated priming effects.

In conclusion, our analysis suggests that rhizosphere priming is an important factor regulating SOM decomposition, the release of plant available N, and ultimately soil C sequestration. As plants tend to increase belowground C allocation with increased temperatures and CO_2_ concentrations, priming effects need to be considered in our long-term analysis of soil C budgets in a changing environment. However, the priming of SOM cannot be viewed in isolation. We also need to consider the ways in which C and N cycling is coupled and constrained by each elements behavior, as the extent of the priming seems to be intimately linked to C and N availability. In such an analysis, it is vital to consider the stoichiometric nutrient demands of the plants and microorganisms involved, and the consequences of that for the way in which they interact.
